# Innovations in the Integrated Management of Breast Cancer

**DOI:** 10.3390/jpm12040531

**Published:** 2022-03-28

**Authors:** Gianluca Franceschini, Alejandro Martin Sanchez, Elena Jane Mason, Riccardo Masetti

**Affiliations:** Multidisciplinary Breast Center, Dipartimento Scienze della Salute della Donna e del Bambino e di Sanità Pubblica, Fondazione Policlinico Universitario A. Gemelli IRCCS, 00168 Rome, Italy; gianlucafranceschini70@gmail.com (G.F.); elenajanemason@gmail.com (E.J.M.); riccardo.masetti@policlinicogemelli.it (R.M.)

Breast cancer is commonly acknowledged as an international priority in healthcare. To date, it is the most common cancer in women worldwide and demographic trends show a steady increase in incidence.

Over the years, increasing efforts and resources have been devoted to a meticulous analysis of risk factors, diagnostic tools and treatment strategies in order to enhance every step of breast cancer management.

Researchers and clinicians strive in search of an optimized, systematic strategy in the diagnosis and treatment of this disease. This effort has led to the creation of the “breast unit model”, which is today considered a gold standard to ensure optimal clinical services centered on patients and based on research through multidisciplinary and integrated management [1]. This approach, involving surgical, radiation and medical oncology, allows the optimization of oncological and cosmetic outcomes and the prolonged survival and improvement of patient quality of life; the integrated treatment is tailored to each patient and based on clinical examination, patient status, disease staging, biologic phenotype such as hormone receptor status and human epidermal growth factor receptor 2 (HER2) overexpression, and patient preferences. The decision-making process in the management of breast cancer includes a detailed discussion with the patient about the risks and benefits associated with the selected treatment.

This Special Issue highlights many recent innovations in the integrated management of breast cancer, their potential advantages and the many open issues that still wait to be properly defined and addressed. The authors’ interests span every aspect of breast cancer care: from early breast cancer to metastatic patients, and from surgical assessment to artificial intelligence application in data collection.

Cancer biology is addressed in two pre-clinical studies analyzing breast tissue samples. Santandrea et al. focus on hormone receptor expression in normal breast tissue, in search of a pattern that could favor the development of a breast tumor [2], while a study by Fuso et al. examines breast cancer patients treated with neoadjuvant chemotherapy in search of a miRNA expression associated with survival, and therefore acting as a predictive biomarker in women affected by early breast cancer [3].

An accurate and comprehensive preoperative assessment is crucial in order to prepare the patients for surgery, and breast cancer care still holds many issues waiting to be fine-tuned. Nonpalpable lesions can compromise and delay an otherwise smooth operation, and the surgeon should be well-prepared with potential solutions to this common problem. This Special Issue offers a review of current image-guided techniques, highlighting the benefits and controversies of each method [4]. Radiology is also tackled in a study focusing on the best imaging technique to assess patients scheduled to receive breast reconstruction via a DIEP flap, and the researchers advocate conventional CT as an alternative to the traditional but costly CT angiography [5].

During the last decade, the goal in surgery has been to make procedures less and less invasive. Much like breast surgery, which has witnessed a gradual diffusion of breast conserving techniques, axillary surgery has also evolved in an increasingly conservative manner. Where previous surgical approaches tended to favor axillary dissection at all costs, the introduction of sentinel lymph node biopsy (SLNB) has led to the preservation of non-pathological axillary lymph node tissue, and once frequent complications such as post-operative lymphedema have greatly diminished in recent years [6]. In this Special Issue we explore the possibilities of a further evolution in axillary surgery, where treatment with sole SLNB could be extended to include patients downstaged to ycN0 by neoadjuvant chemotherapy [7].

When a breast-conserving approach cannot guarantee both adequate local control and a good aesthetic result, the surgeon has to perform a mastectomy. Innovative surgical procedures called “conservative mastectomies” with immediate prepectoral implant reconstruction have been introduced in order to obtain more favorable aesthetic outcomes and avoid problems caused by manipulation of the pectoralis major muscle, such as breast animation deformity, postoperative pain and injury-induced muscular deficit [8].

The primary goal of management in metastatic disease is the alleviation of symptoms, maintenance or improvement in quality of life and prolongation of survival despite possible treatment toxicity. Patients with metastatic disease receive systemic medical treatments including endocrine therapy, chemotherapy, biologic therapies, targeted and immunotherapy and supportive care measures. However, a subset of patients may benefit from a specific loco-regional treatment [9]: oligometastatic disease has been the object of particular interest because of the possibility to aim for a long-term remission in these patients, and once-discarded options such as liver metastasectomy have been shown to be a possible therapeutic option in selected patients [10,11].

The benefits of a multimodal prehabilitation model are emerging in recent studies, as in this framework patients may be more receptive to health behavior changes in a structured support network. Di Leone et al. shed light on a possible personalized prehabilitation model to enhance patient care in the neoadjuvant setting, which allows each patient to receive the attention of every required specialist in a set frame of time [12,13]. For example, elderly patients can greatly benefit from a preoperative geriatric assessment in order to avoid negative outcomes deriving from otherwise unknown syndromes such as severe sarcopenia [14]. On the other hand, younger women with a new, unexpected diagnosis of breast cancer may face issues related to sexuality and fertility, and studies addressing the impact of treatment on ovarian reserve are paramount to better understand the mechanisms leading to early menopause and subsequent infertility. The clinician’s primary objective is to offer a timely oncofertility service, in order to preserve the opportunity for family planning without delaying chemotherapy [15]. Similar strategies must be adopted when confronting pregnancy-associated breast cancer, a rare occurrence that nonetheless threatens the wellbeing of both mother and fetus [16].

Finally, the last few years have seen the creation of new artificial intelligence technologies with the potential to radically change the modern management of breast cancer. Research itself is a viable candidate for the coming high-tech revolution: today, protocol development can be promoted, patient enrollment can be enhanced by a patient-trial matching made possible by the growing diffusion of electronic health records, and patient parameters and adherence to trials can be monitored in real-time by a variety of wearable devices. This Issue witnesses the transformation, thanks to the contribution of authors active in the field of real-world data: Cesario et al. describe the development of a digital research assistant that manages patient enrollment in trials with the employment of an artificial intelligence algorithm [17], while Marazzi et al. exploit text mining to successfully extract data from heterogeneous sources and to generate clinical evidence [18,19].

This Special Issue finds its place in the modern panorama of breast care by promoting a modern, holistic approach to breast disease and encouraging clinicians to tailor patient treatment. The development of appropriate clinical pathways, with a multidisciplinary and standardized approach, is essential for successful, well-rounded treatment in the era of personalized medicine.
